# Undifferentiated Bronchial Fibroblasts Derived from Asthmatic Patients Display Higher Elastic Modulus than Their Non-Asthmatic Counterparts

**DOI:** 10.1371/journal.pone.0116840

**Published:** 2015-02-13

**Authors:** Michal Sarna, Katarzyna A. Wojcik, Pawel Hermanowicz, Dawid Wnuk, Kvetoslava Burda, Marek Sanak, Jarosław Czyż, Marta Michalik

**Affiliations:** 1 Department of Medical Physics and Biophysics, Faculty of Physics and Applied Computer Science, AGH University of Science and Technology, Krakow, Poland; 2 Department of Cell Biology, Faculty of Biochemistry, Biophysics and Biotechnology, Jagiellonian University, Krakow, Poland; 3 Department of Pharmaceutical Biochemistry, Faculty of Pharmacy, Jagiellonian University Medical College, Krakow, Poland; 4 Department of Plant Biotechnology, Faculty of Biochemistry, Biophysics and Biotechnology, Jagiellonian University, Krakow, Poland; 5 Laboratory of Molecular Biology and Clinical Genetics, Medical College, Jagiellonian University, Krakow, Poland; LAAS-CNRS, FRANCE

## Abstract

During asthma development, differentiation of epithelial cells and fibroblasts towards the contractile phenotype is associated with bronchial wall remodeling and airway constriction. Pathological fibroblast-to-myofibroblast transition (FMT) can be triggered by local inflammation of bronchial walls. Recently, we have demonstrated that human bronchial fibroblasts (HBFs) derived from asthmatic patients display some inherent features which facilitate their FMT *in vitro*. In spite of intensive research efforts, these properties remain unknown. Importantly, the role of undifferentiated HBFs in the asthmatic process was systematically omitted. Specifically, biomechanical properties of undifferentiated HBFs have not been considered in either FMT or airway remodeling *in vivo*. Here, we combine atomic force spectroscopy with fluorescence microscopy to compare mechanical properties and actin cytoskeleton architecture of HBFs derived from asthmatic patients and non-asthmatic donors. Our results demonstrate that asthmatic HBFs form thick and aligned ‘ventral’ stress fibers accompanied by enlarged focal adhesions. The differences in cytoskeleton architecture between asthmatic and non-asthmatic cells correlate with higher elastic modulus of asthmatic HBFs and their increased predilection to TGF-β-induced FMT. Due to the obvious links between cytoskeleton architecture and mechanical equilibrium, our observations indicate that HBFs derived from asthmatic bronchi can develop considerably higher static tension than non-asthmatic HBFs. This previously unexplored property of asthmatic HBFs may be potentially important for their myofibroblastic differentiation and bronchial wall remodeling during asthma development.

## Introduction

Bronchial asthma is one of the most common chronic diseases throughout the world and its incidence has been continuously increasing in recent decades. Extensive airway remodeling observed during asthma is related to local airway inflammation and the concomitant epithelial damage and thickening of bronchial walls [1]. Asthma progression is thought to depend on hyperplasia, hypertrophy and phenotypic transitions of bronchial epithelial cells, smooth muscle cells and fibroblasts. These processes are accompanied by increased extracellular matrix (ECM) deposition in asthmatic bronchi [2] and are the fundamental factors associated with clinical symptoms of asthma, in particular with airway constriction [1]. Discrete myofibroblastic lineages are also thought to participate in the formation of sub-epithelial fibrosis and bronchial wall remodeling [3–4]. They develop in asthmatic bronchi mainly as a consequence of pathologic TGF-β—induced fibroblast-to-myofibroblast transition (FMT). Myofibroblasts display phenotypes similar to smooth muscle cells, with *de novo* expression of α-smooth muscle actin (α-SMA) and its incorporation into highly contractile microfilament bundles (stress fibers) [5].

The architecture of the actin cytoskeleton, affected by the quality and quantity of cell adhesion to ECM and by the contractile activity of actomyosin, determines the mechanical properties of myofibroblasts and fibroblasts [6–7]. The abundance, alignment and thickness of stress fibers and the size of focal adhesions also reflect the orientation and magnitude of static forces exerted by these cells on their microenvironment [8–9]. A cell can increase its stress state by contracting and applying mechanical load to itself in the process of auto-loading [10–11]. Concomitantly, intercellular contacts and/or ECM mediate mechanical interactions of the cells in niches [12]. Physical stimuli not only modulate mechanical properties of the cells but can also regulate their phenotype via compartmentalisation of signaling pathways responsive to paracrine signals [13–14]. For example, mechanical equilibrium of bronchial fibroblasts (cultured on substrates with varying stiffness) has been shown to determine their susceptibility to TGF-β—induced FMT [12]. Accordingly, interrelations have been postulated between the properties of ECM that constitutes bronchial walls, mechanochemical properties of undifferentiated fibroblasts and pathological FMT. However, the role of mechanical activity of undifferentiated bronchial fibroblasts in asthma development and in bronchial wall remodeling remains unknown.

The presence of prominent stress fibers in undifferentiated HBFs derived from asthmatic patients *in vitro* [15] indicates their high intrinsic contractile activity [6]. We have also demonstrated the predilection of these cells for TGF-β—induced FMT [16–17]. Airway remodeling is ascribed *inter alia* to the increased contractility of myofibroblasts and smooth muscle cells [18–19]. However, the magnitude of static tension developed by undifferentiated fibroblasts from asthmatic and non-asthmatic bronchi has not yet been compared. Furthermore, it remains an open question whether auto-loading processes play any role in the regulation of HBF capacity for myofibroblastic differentiation. Atomic force microscopy (AFM) analysis is frequently used for monitoring changes in the elasticity of cells derived from patients with different diseases, with a particular emphasis on cancer cells [20–21]. Due to the obvious links between elasticity and mechanical equilibrium of the cells, this technique can be used to correlate different systemic disorders with isometric tension and other biomechanical properties of the cells [22–23]. To achieve this aim with regard to bronchial wall remodeling in asthma, we performed comparative cytometric and an AFM study of the cytoskeleton architecture and mechanical properties of HBFs derived from asthmatic (AS) and non-asthmatic (NA) biopsies, cultured on solid substratum.

## Materials and Methods

### Subjects

The study was performed on two groups of subjects. The first group consisted of four non-asthmatic individuals (4 samples; NA group) in whom diagnostic bronchoscopy ruled out any serious airway pathology, including asthma, fibrotic lung disease, sarcoidosis, and cancer. The second group consisted of four patients with diagnosed moderate asthma (4 samples; AS group). The clinical characteristics of the groups of patients are shown in Table 1. All patients were treated in the Department of Medicine of Jagiellonian University and were in stable clinical condition. The study was approved by the University’s Ethics Committee (KBET/211/B/2013) and all the patients provided written consent to participate in the study.

**Table 1:** Characteristics of study participants.

**Subjects**	**Non-asthmatic (NA)**	**Asthmatic (AS)**
Number of subjects	4	4
Age (year), mean, range	75 (65–88)	54 (44–66)
Sex (male/female)	2/2	3/1
Duration of the disease (years)^a^	-	12.5±7.3
FEV1 (% of predicted)^a^	103.4±12.6	75.8±28.8
Use of inhaled steroids (treated subjects/all subjects)	0/4	4/4
Use of systemic steroids (treated subjects/all subjects)	0/4	0/4

^a^Data presented as means with standard deviation

### Sample preparation

Bronchial biopsies were obtained from the segmental bronchi during bronchoscopy using a fiberoptic bronchoscope (Olympus, Japan). Primary human bronchial fibroblasts (HBFs) were isolated as described previously [16, 24]. Briefly, immediately after isolation the submucosal biopsy specimens were put into a tube containing cold PBS (Sigma) for 20 minutes and then transferred into DMEM culture medium (Sigma-Aldrich, St. Louis, MO, USA) with 10% FCS (Gibco BRL) containing 1 mg/mL of collagenase type I (Gibco BRL). After 4 to 6 hours of incubation at 37°C, the digested parts of the biopsies were harvested by centrifugation (5 minutes at 90g). Cells were seeded in 6-well plates (Falcon, Primaria, Becton Dickinson) and cultured in DMEM supplemented with 20% FCS, antibiotics and antimycotics for about 2 weeks. The medium was changed every 24 hours up to the day when the primary cultures of HBFs reached about 80% of confluence. Then the cells were passaged onto new plates using a standard trypsinisation protocol.

For secondary cultures, DMEM supplemented with 10% FCS was used. After isolation, the cells were inspected to confirm that they had fibroblast-like morphology. The levels of expression of α-SMA, desmin and vimentin were checked by immunofluorescence staining. In all primary cultures maintained under standard conditions (cultured medium with 10% FCS), less than 5% of cells exhibited a very weak stain for α-SMA, no cells stained for desmin and all cells expressed vimentin. These results confirmed that the samples were not contaminated by smooth muscle cells. Cells between the 5th and 10th passage were used in the experiments.

### Fluorescence microscopy analysis

Analyses of actin cytoskeleton architecture and vinculin localization in HBFs were made on samples fixed with 3.7% formaldehyde, permeabilised with 0.1% Triton X-100 and blocked with 3% bovine serum albumin. Specimens were immunostained with mouse monoclonal anti-human vinculin IgG (Sigma-Aldrich) and Alexa Fluor 488-conjugated goat anti-mouse IgG (clone A11001, Sigma-Aldrich), and counterstained with TRITC-phalloidin (Sigma-Aldrich). Specimens were analysed with a Leica DMI6000 B inverted microscope (Leica Microsystems GmbH, Wetzlar, Germany). Analyses of intracellular vinculin localization were performed using Total Internal Reflection Fluorescence (TIRF). Numbers and lengths of vinculin^+^ FAs were estimated with LAS AF Software (Leica Microsystems GmbH, Wetzlar, Germany). Taking into consideration the optical resolution of the system, elongated (>1μm in length) FAs adjacent to stress fiber termini were analyzed. 5–7 representative cell images from each AS and NA sample (n = 25 for AS and NA groups, respectively) were analyzed.

### AFM analysis

For AFM analysis, cells were seeded onto Petri dishes at a density of 1000 cells/cm^2^ and incubated overnight in DMEM supplemented with 10% FCS, at 37°C in a 5% CO_2_ humidified atmosphere. AFM analyses were conducted using an Agilent 5500 atomic force microscope (Agilent Technologies, Austin, Texas, USA) and carried out in culture medium at 37°C. Optical preview was used during the measurements to ensure that the analyses were performed on cells displaying typical “fibroblastic” morphology. Mechanical analysis was performed in force spectroscopy mode [25]. Force measurements were collected using standard silicon nitride cantilevers (Bruker Probes, Camarillo, California, USA) with a nominal tip radius of 20 nm. Calibration of the spring constant of the probes was made before and after mechanical analysis of cells using the thermal tune procedure as described elsewhere [26]. Curves from 10 to 30 randomly selected points were collected from the central region of the cell at a rate of 1 Hz. 10 force curves were measured at each point for statistical analysis. To prevent any damage to the cells, measurements were carried out in the range of forces selected to obtain shallow indentations of the cells (<400 nm). Forces exerted on the cells during the analysis varied between 0.1 nN and 1 nN for NA and AS HBFs, respectively. A total number of 30 cells was investigated for each sample. Before each cell was measured, its topography was imaged using tapping mode to precisely localize the central region of a cell for mechanical analysis. To show the co-localization effect of stress fibers and their impact on the elastic properties of the cells, representative force maps of the cells were made. Force maps were obtained from a grid of 128 × 128 force curves applied onto the scan area. Forces were selected to obtain high levels of cell deformation during this procedure. The values of the Young’s modulus were estimated from force curves by converting force-displacement curves into force-indentation curves and fitting them with the modified Hertz model [27]. To avoid possible substrate induced effects, the correction of thin samples was used during the analysis of force curves as described elsewhere [28]. A detailed description of the analysis of force curves used in this study and the force tomography approach can be found elsewhere [29]. The half opening angle of the AFM tip was 25° and the Poisson ratio of the cells was taken to be 0.5, which is typical for soft biological materials [30].

### Western Blotting

HBFs and human cardiac mesenchymal stromal cells (hcMSC; kindly provided by Ewa Zuba–Surma from the Department of Cell Biology, Faculty of Biochemistry, Biophysics and Biotechnology, Jagiellonian University) were cultured in Petri dishes in a medium supplemented with 10% FCS for 24 hours, or in serum-free medium supplemented with 5ng/ml TGF-β_1_ for 7 days (AS HBFs only), respectively. Afterwards, immunoblots were performed as described previously [15]. In brief, cells were lysed and 10 μg of protein from whole cells lysates were separated by 15% SDS-polyacrylamide gel electrophoresis and then transferred into polyvinylidene difluoride membrane (Hybond-P, Amersham Pharmacia Biotech). After blocking, membranes were incubated with mouse monoclonal antibody (Sigma-Aldrich): anti-actin IgG (clone AC-40, 1:1000), anti-vinculin IgG (clone hVIN-1, 1:200), anti-α-SMA IgG (1:1000), anti-desmin IgG (clone DE-U-10, 1:100), anti-vimentin IgM (clone VIM-13.2, 1:500) or anti-GAPDH IgM (clone GAPDH-71.1, 1:3000), respectively. After washing 3 times, the membrane was incubated with the relevant secondary antibody (goat-anti mouse IgG and goat-anti mouse IgM, respectively) conjugated with horseradish peroxidase (1:3000, Invitrogen, Carlsbad, CA). The detection of bands was done using Super Signal West Pico Substrate (Pierce, Rockford, IL) and the MicroChemii imaging system (SNR Bio-Imaging Systems, Jerusalem, Israel). The relative optical density (ROD) was estimated from 4 samples belonging to AS and NA groups, respectively, using ImageJ 1.45s software.

### Analysis of cell motility

Cell movement was recorded with a Leica DMI6000B time-lapse system equipped with a temperature/CO_2_ chamber, IMC contrast optics and a cooled, digital DFC360FX CCD camera as described elsewhere [32]. Analyses were carried out in culture medium at 37°C and in 5% CO_2_ atmosphere. Motility of individual cells was recorded for 10 hours, with 15 minute time intervals. Trajectories of individual cells were determined from a series of changes in the cell centroid positions with the Hiro program (written by W. Czapla) as described previously [31–32]. The following parameters were quantified: (i) total length of the cell trajectory (μm); i.e. the trajectory of a cell considered as a sequence of 40 straight-line segments, each corresponding to cell centroid translocation within one time interval between two successive images; (ii) the total length of the cell displacement (μm); i.e. the distance from the starting point directly to the cell’s final position.

### Statistical analysis

Statistical significance of differences in mean values was assessed using the two-sample independent Student’s t-test (AFM, immunofluorescence) and non-parametric Mann-Whitney U-test (motility) at the 95% confidence level. Differences among means are reported using approximated P values. Statistical analysis was made using Mathematica 8.0 software.

## Results and Discussion

### AS HBFs develop more prominent stress fibers and matured focal adhesions than their NA counterparts

**Fig 1:**
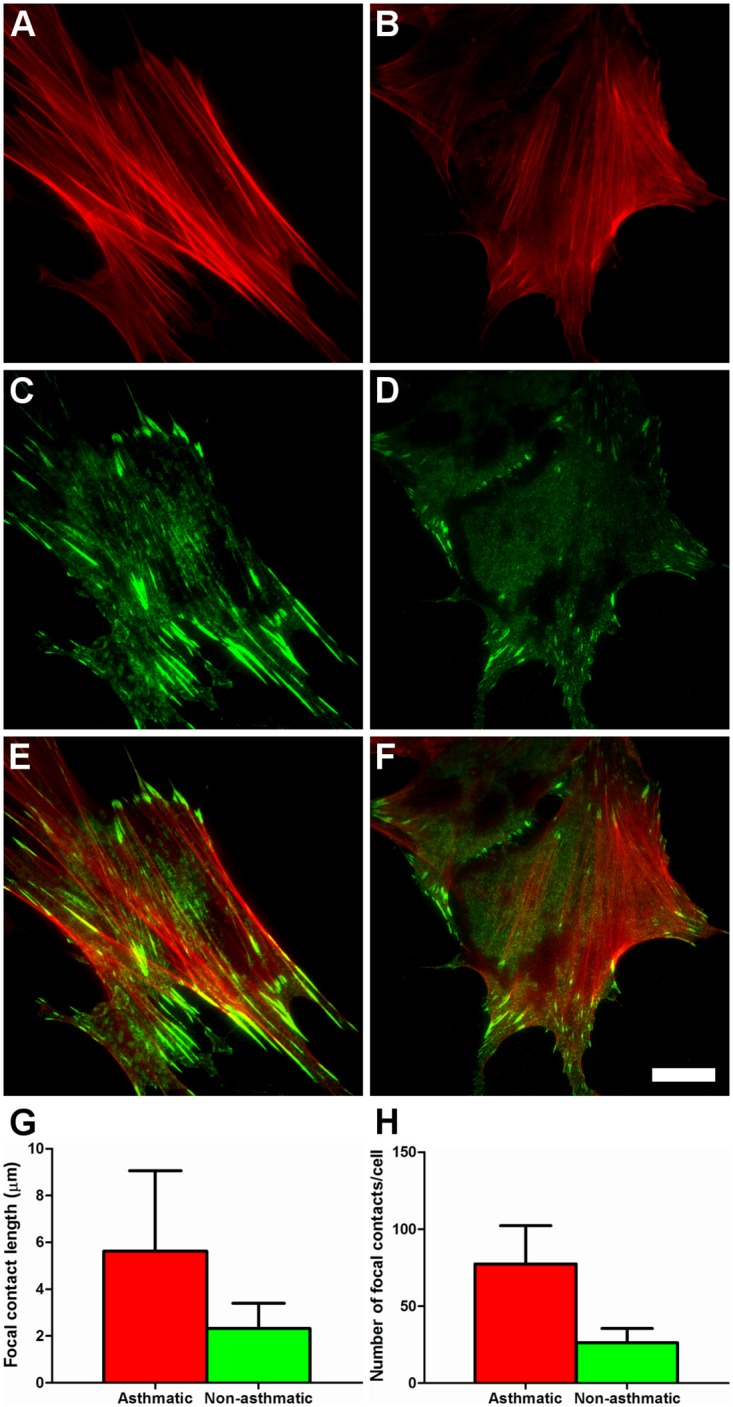
Actin cytoskeleton organization in AS and NA HBFs analyzed by fluorescence microscopy. Representative images of HBFs stained against F-actin (A, B) and vinculin (C, D) show that HBFs from the AS group (A, C) develop more prominent F-actin^+^ stress fibres and vinculin^+^ FAs than their NA counterparts (B and D, respectively). (E, F) depict overlay images of F-actin and vinculin staining. Scale bar represents 20 μm. (G, H) summarize the results of semi-quantitative analyses of FA lengths and numbers in AS and NA HBFs which confirmed the differences in the architecture of the actin cytoskleton between cells belonging to both analyzed groups. Values are the means ± s.d. for each group compiled from 4 AS and 4 NA samples, respectively). See S1 Fig. for data on individual samples.

We have shown previously that HBFs derived from asthmatic patients develop prominent “ventral” stress fibers [15, 17]. These microfilament bundles lay along the base of the cells and are anchored to ECM through vinculin-rich focal adhesion complexes (FAs) at each end [9]. The function of stress fibers *in vivo* is still a matter of debate [11]. However, stress fibers anchored to ECM through FAs contract isometrically [9], meaning that they generate tension without the shortening of their lengths [33–34]. Therefore, stress fibers illustrate the magnitude of cellular auto-loading and direction of mechanical forces exerted by a cell on the substratum [8]. Fluorescence microscopy analysis of the actin cytoskeleton revealed that AS HBFs had significantly more ‘pronounced’ stress fibers than NA cells (Fig. 1A–B; see S1 Fig. for data on AS and NA HBF samples). Accordingly, the visualization of FAs in these cells by TIRF microscopy revealed significant differences in the sizes of vinculin-rich FAs between HBFs from AS and NA groups (Fig. 1C, D; see Fig. 1E, F for the co-localization of stress fibers and FAs). FAs in AS HBFs were much larger than those of NA cells (Fig. 1G). Their lengths in AS and NA HBFs ranged from 1.0 μm to 27.1 μm, and from 1.0 μm to 9.1 μm, respectively. Average FA length (mean ± s.d.) was 5.62 ± 3.44 μm (n = 1620) for AS HBFs and 2.32 ± 1.08 μm (n = 655) for NA HBFs. Moreover, the number of FAs per cell was significantly higher in HBFs from the AS (77.32 ± 24.88) than from the NA group (26.16 ± 9.25) (Fig. 1H). Differences in the cytoskeleton architecture between AS and NA HBFs observed in this study suggest that the magnitude of isometric tension in undifferentiated AS HBFs is considerably higher than in NA cells.

### AS HBFs display higher elastic modulus than NA HBFs

**Fig 2:**
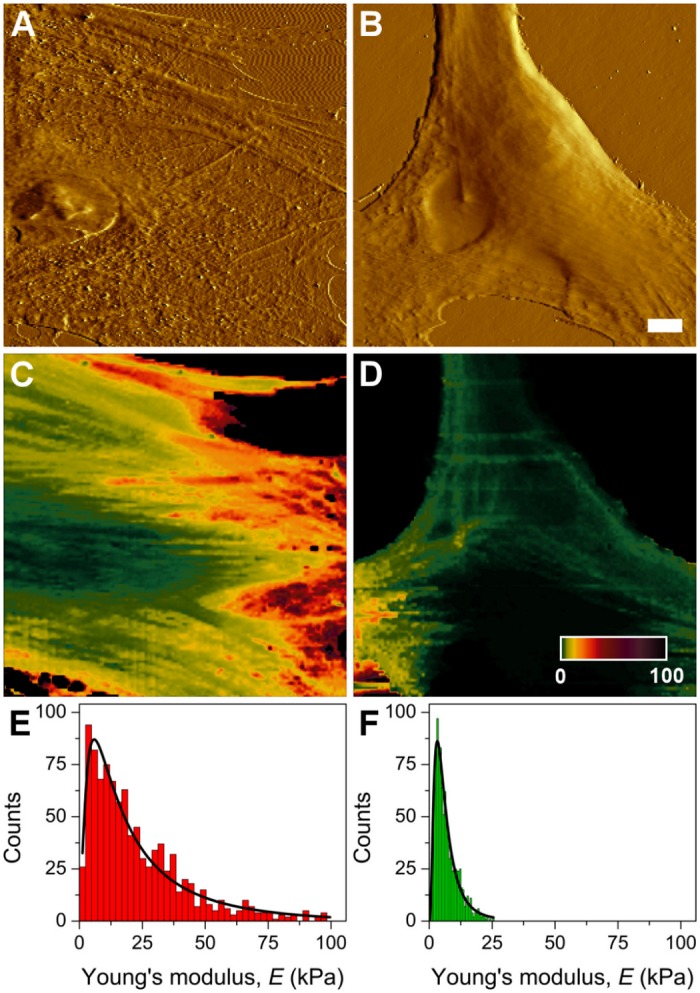
Atomic force microscopy analysis of the morphology and mechanical properties of AS and NA HBFs. Representative AFM amplitude images of HBFs from AS (A) and NA groups (B) show differences in the morphology between the cells. Stress fibers in the case of AS cells are much more pronounced when compared to NA cells. Scale bar represents10 μm. Force maps of AS (C) and NA cells (D) show significant differences in the values of the Young’s modulus between AS and NA HBFs. Color scale bar in force maps represents the values of the Young’s modulus given in kPa. All collected nanomechanical data belonging to AS and NA groups, are summarized in the histograms of the Young’s modulus values (compiled from 4 AS (E) and 4 NA samples (F), respectively). Log-normal fit was applied to the data to determine the average values of the Young’s modulus for each group. Counts on the y axes represent mean values of E calculated from 10 force curves measured at each location on a single cell. A total number of 120 cells were investigated for each group. Mechanical data for individual samples is shown in S2 Fig.

Atomic force microscopy analyses confirmed differences in the mechanical equilibrium between AS and NA HBFs. Elasticity measurements revealed significant differences in the values of the Young’s modulus (E) between HBFs derived from AS and NA groups (Fig. 2). Data collected from all analyzed AS and NA HBFs (4 NA; n = 961 and 4 AS; n = 1076) yielded average E values (mean ± s.d.) of 18.75 ± 6.78 kPa and 5.39 ± 0.97 kPa, for AS and NA cells, respectively. A two-sample independent t-test showed that these values were significantly different from each other at the 95% confidence level (P < 0.0001). The distribution of Young’s modulus for AS cells was significantly higher than that of NA cells. The obtained E values for HBFs from AS group were in the range of 1.05 ± 0.02 to 97.91 ± 1.53 kPa, whereas the E values for HBFs from NA group were in the range of 0.71 ± 0.14 to 25.49 ± 0.41 kPa (Fig. 2E, F). Correspondingly, the average value of the Young’s modulus (mean ± s.d.) of HBFs from single AS samples were in the range of 16.07 ± 9.32 to 22.32 ± 5.52 kPa (S2A, C, E and G Fig.), whereas the average value of the Young’s modulus (mean ± s.d.) of the single HBF samples from the NA group was in the range of 3.79 ± 0.88 to 7.49 ± 2.61 kPa (S2B, D, F and H Fig.).

The results indicate that AS HBFs have on average nearly four times higher elastic modulus than NA HBFs. These observations confirm elevated isometric tension of AS HBFs *in vitro*. The elastic properties of sub-membranous cortex that were actually measured by AFM are determined by the mechanical equilibrium of the underlying actin cytoskeleton [35]. On the other hand, a correlation between the stiffness of single stress fibers and their tension has been reported [36]. Thus, it can be hypothesized that differences in cell cortex elasticity observed between AS and NA cells are related to the differences in the mechanical balance of stress fibers.

### Increased susceptibility of undifferentiated AS HBFs to TGF-β_1_-induced FMT correlates with their increased elastic modulus

**Fig 3:**
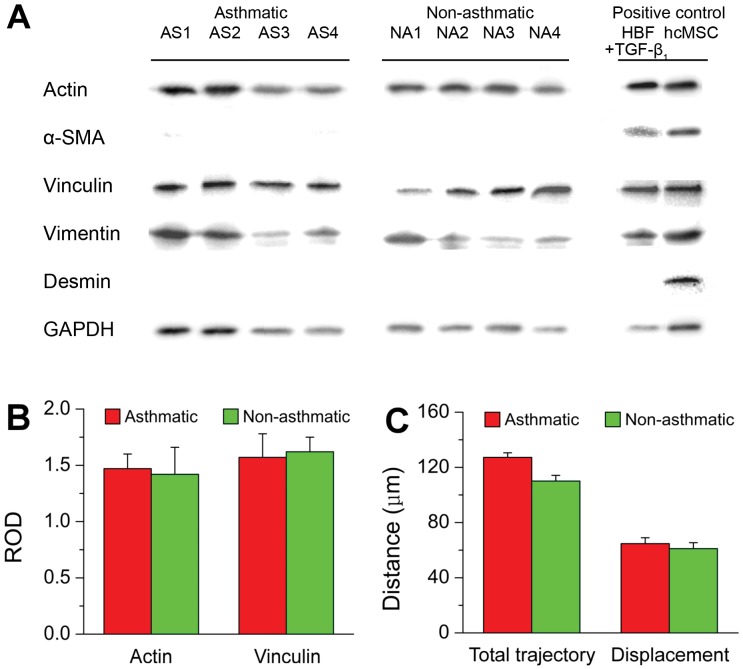
Gene expression profile and motility of AS and NA HBFs. A lack of α-SMA (myofibroblast marker) and desmin (muscle cell marker) expression in HBF samples from AS and NA groups (A) was accompanied by a lack of significant differences in the total expression of actin and vinculin between AS and NA samples, as shown by densitometric analysis (B). Human cardiac mesenchymal stromal cells (hcMSC) and TGF-β_1_-stimulated AS HBFs were used as positive controls for desmin and α-SMA, respectively. Relative optical density (ROD) values represent vinculin and actin levels (mean ± s.d.) calculated for each group by compilation of 4 AS and 4 NA samples, respectively, normalized against GAPDH levels. Time-lapse analyses of cell locomotion did not reveal any significant differences in the motile activity (the total length of displacement and the total length of trajectory) between AS and NA cells (C). Values are means ± s.d. for each group compiled from 4 AS and 4 NA samples, respectively. (*) P < 0.05.

It should be emphasized that HBFs were cultivated for relatively short periods of time (16–22 hours) before immunofluorescence and AFM analyses. This limited the influence of ECM deposition on mechanochemical properties of the cells. Moreover, immunoblot analyses of AS and NA HBF populations did not reveal any significant differences in total actin and vinculin levels between the analyzed HBF groups (Fig. 3A, B). A lack of detectable α-SMA and desmin expression in analyzed cells demonstrated that HBFs were undifferentiated and also excluded the presence of smooth muscle cells in the examined samples (Fig. 3A). Moreover, the analyses of cell movement showed no significant differences in the motile activity between AS and NA HBFs (Fig. 3C). This observation is of importance because cell motility can affect the stability of cell adhesion and the size and intracellular distribution of FAs.

On the other hand, we observed a correlation between the elastic properties of HBFs and their susceptibility to TGF-β_1_-induced FMT. Table 2 summarizes data on mechanical properties and on the efficiency of FMT of HBFs in all tested NA and AS samples. Cells derived from AS patients yielded TGF-β_1_—stimulated FMT efficiency to be approximately 70%, whilst only 7% of the cells from NA individuals underwent FMT in the corresponding conditions. The results of this study confirm the functional links between the nanomechanics of fibroblasts and FMT reported elsewhere [12] and indicate that FMT efficiency may be related to the mechanochemical properties of undifferentiated HBFs. These data are consistent with our previous work which demonstrated that HBFs derived from patients with diagnosed asthma displayed unknown features *in vitro* that facilitated their FMT [16, 37–38]. They may confirm the role of mechanical balance of the actin cytoskeleton in the regulation of TGF-β signaling [4, 39]. Cytoskeleton-dependent outside-in and inside-out signaling is crucial for cell adhesion, migration, proliferation, differentiation and apoptosis in general [40–42], and the function of lung fibroblasts in particular [12]. Mature FAs observed in AS HBFs may participate in collaborative signaling triggered by TGF-β, thus determining cell reactivity to this paracrine factor. On the other hand, our experiments were done on samples consisting of “undifferentiated” cells. Therefore, the analyzed NA and AS HBFs might represent functionally discrete lineages, in spite of similar phenotypes (lack of α-SMA expression). In asthmatic bronchi, highly contractile α-SMA-positive myofibroblasts can differentiate from stress fiber-containing but α-SMA-negative fibroblasts and/or proto-myofibroblasts [5]. Both phenotypes can co-exist *in vivo* and perform different functions [6], however the precise role of proto-myofibroblasts in asthma remains unknown. Their susceptibility to TGF-β_1_ stimulation finally determines the abundance of the myofibroblastic fraction in the bronchial wall [1], therefore further studies are necessary to infer whether AS HBFs actually represent a ‘contractile’ proto-myofibroblastic phenotype.

**Table 2:** Patient diagnosis versus mechanical analysis (the Young’s modulus, *E*) compared with FMT efficiency of the cells.

**Case no.**	Clinical diagnosis of patients*	**Elastic modulus (kPa)^†^**	**FMT efficiency^‡^**
1	Asthmatic	16.07±9.32	79%
2	Asthmatic	18.69±6.99	67%
3	Asthmatic	19.99±7.58	59%
4	Asthmatic	22.32±5.52	73%
5	Non-asthmatic	3.79±0.88	7%
6	Non-asthmatic	4.33±0.58	6%
7	Non-asthmatic	7.44±1.09	7%
8	Non-asthmatic	7.49±2.61	7%

*Clinical diagnosis was made based on a routine asthma examination.

^†^Elastic modulus values (E) represent mean ± s.d.

^‡^FMT efficiency was measured based on the analysis of the number of cells (%) expressing and incorporating into stress fibers α-SMA after stimulation with TGF-β1 *in vitro*. These results are in agreement with data published previously (16–17).

## Conclusions

In asthmatic bronchi, the contractile activity of smooth muscle cells and myofibroblasts is considered to induce the remodeling of bronchial walls [18–19] in which undifferentiated fibroblasts are embedded. Previous studies have suggested that TGF-β_1_-treated fibroblasts containing α-SMA^+^ stress fibers are characterized by enhanced contractility and generate relatively high traction forces [6, 43]. Independently, isometric tension related to the contractility of the cells residing in bronchial walls can facilitate their TGF-β_1_- induced myofibroblastic differentiation [12]. Combined nanomechanical and cytofluorimetric analyses *in vitro*, performed in this study, revealed high isometric tension of undifferentiated (α-SMA—negative) HBFs derived from bronchial biopsies of asthmatic patients. Extrapolating these data to the *in vivo* situation, we propose that higher tension exerted by HBFs on surrounding tissue in asthmatic bronchi can affect their susceptibility to TGF-β—induced FMT and, concomitantly, contribute to bronchial wall remodeling. Our observations point to the possible role of the mechanical activity of undifferentiated HBFs for asthma progression *in vivo*. The results of this study, together with the existing knowledge on asthma development, may help to better understand the interrelations between FMT of bronchial cells and the asthmatic process. This could potentially improve the effectiveness of current treatment regimens of this disease.

## Supporting Information

S1 Fig Actin cytoskeleton organization in AS and NA HBF samples analyzed by fluorescence microscopy. AS and NA HBFs were cultured in DMEM with 10% FCS for 24 hours and then F-actin was detected with TRITC-phalloidin. Representative images of F-actin staining in AS (A, C, E, G) and NA (B, D, F, H) HBF samples show a more prominent incorporation of F-actin into stress fibers in AS HBFs when compared to their NA counterparts. Scale bar represents 20 μm.
(TIF)

S2 Fig Atomic force microscopy analysis of mechanical properties of HBFs from individual AS and NA samples. Histograms of the Young’s modulus values (E) determined for HBF populations constituting 4 AS and 4 NA samples. The data confirms significant differences between the AS and NA groups. AS cells samples have much higher elastic modulus when compared to NA cells Log-normal fit was applied to all samples to determine the average values of the Young’s modulus for each sample. Counts on the y axis represent mean values of E calculated from 10 force curves measured at each point on a single cell. A total number of 30 cells were investigated for each sample.
(TIFF)
