# Supplementary figures and images for: Undifferentiated Bronchial Fibroblasts Derived from Asthmatic Patients Display Higher Elastic Modulus than Their Non-Asthmatic Counterparts
Source: PLoS One. 2015 Feb 13;10(2):e0116840. doi: 10.1371/journal.pone.0116840 (PMC4334506; doi:10.1371/journal.pone.0116840)

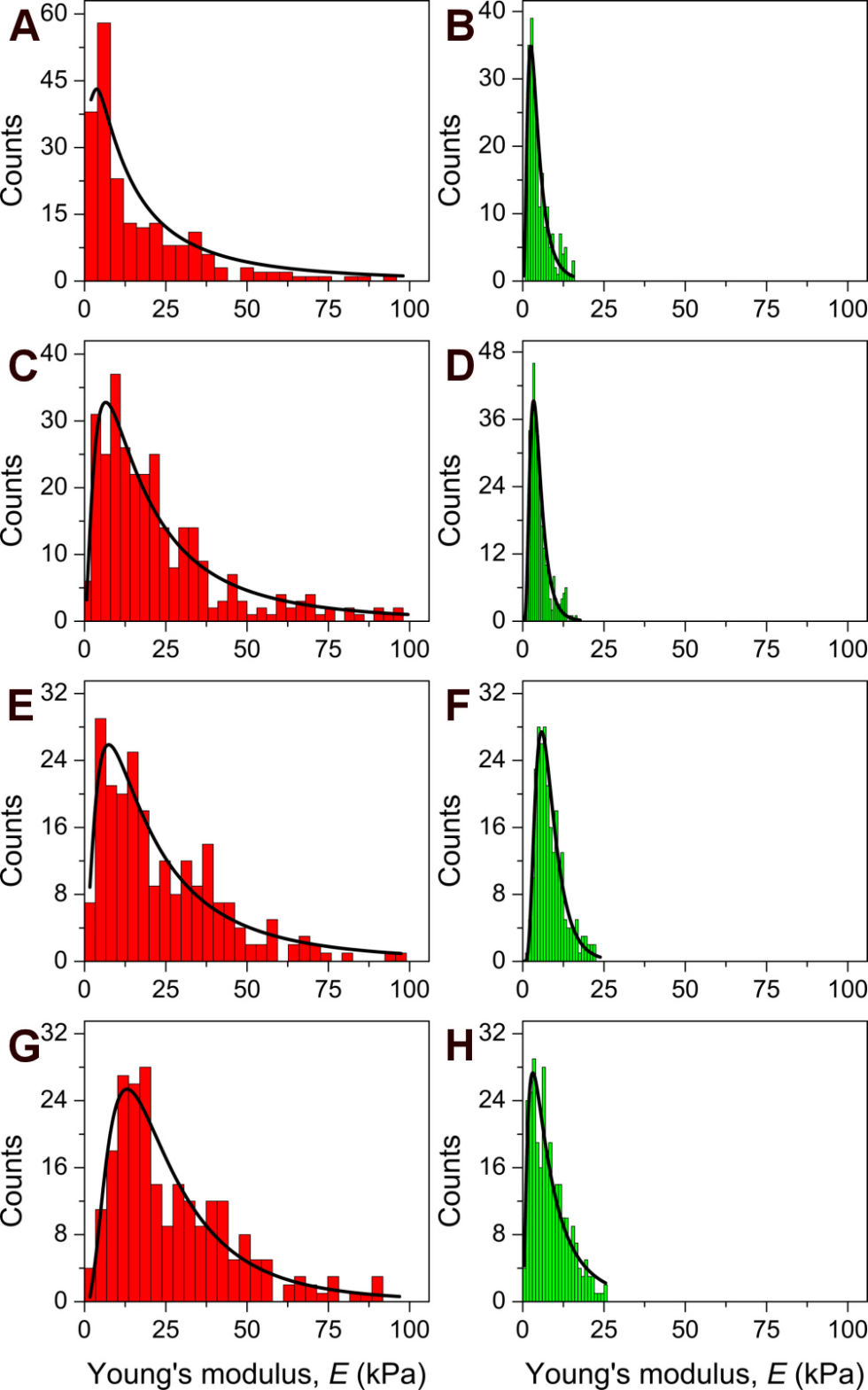

Supplement: S1 Fig — AS and NA HBFs were cultured in DMEM with 10% FCS for 24 hours and then F-actin was detected with TRITC-phalloidin. Representative images of F-actin staining in AS (A, C, E, G) and NA (B, D, F, H) HBF samples show a more prominent incorporation of F-actin into stress fibers in AS HBFs when compared to their NA counterparts. Scale bar represents 20 μm. (TIF) [file pone.0116840.s001.tif]

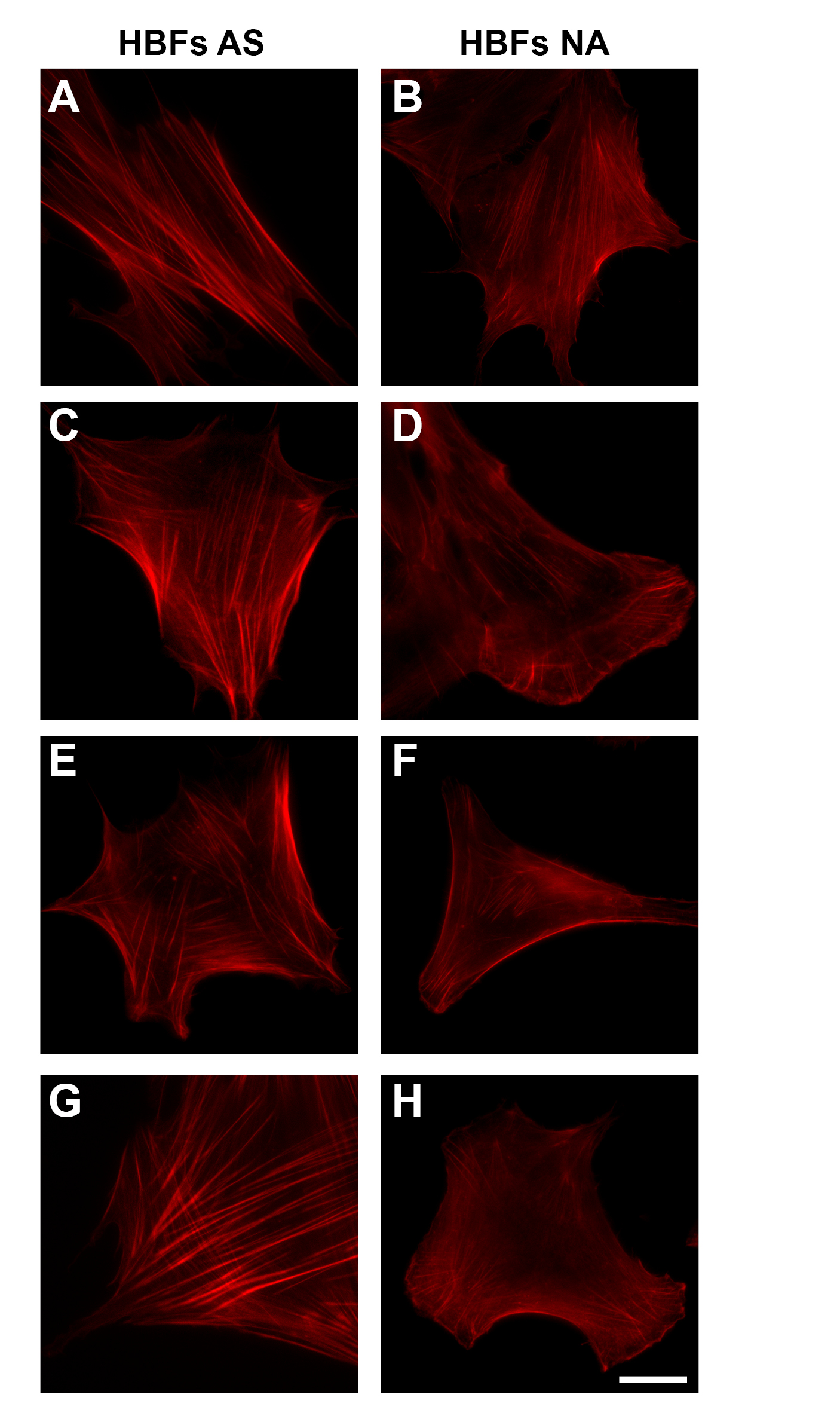

Supplement: S2 Fig — Histograms of the Young’s modulus values (E) determined for HBF populations constituting 4 AS and 4 NA samples. The data confirms significant differences between the AS and NA groups. AS cells samples have much higher elastic modulus when compared to NA cells Log-normal fit was applied to all samples to determine the average values of the Young’s modulus for each sample. Counts on the y axis represent mean values of E calculated from 10 force curves measured at each point on a single cell. A total number of 30 cells were investigated for each sample. (TIFF) [file pone.0116840.s002.tiff]
